# Rapid anticalcification treatment for glutaraldehyde-fixed autologous tissue in cardiovascular surgery

**DOI:** 10.1186/s13019-022-01895-7

**Published:** 2022-05-31

**Authors:** Shotaro Kaneko, Susumu Isoda, Toru Aoyama, Motohiko Goda, Shota Yasuda, Taisuke Shibuya, Mai Matsumura, Hideaki Mitsui, Koji Okudela, Shinichi Suzuki, Daisuke Machida, Munetaka Masuda

**Affiliations:** 1 Department of Surgery, Yokohama City University Hospital, 3-9 Fukuura, Kanazawa-ku, Yokohama, 236-0004 Japan; 2 Cardiovascular Center, Yokohama City University Medical Center, Yokohama, Japan; 3 Department of Pathology, Yokohama City University, School of Medicine, Yokohama, Japan; 4 Department of Cardiovascular Surgery, Fukuoka Wajiro Hospital, Fukuoka, Japan

**Keywords:** Glutaraldehyde, Calcification, Autologous tissue, Cardiovascular surgery

## Abstract

**Background:**

Glutaraldehyde (GA)-fixed autologous tissues, including the pericardium, are widely used as patches and valve substitutes in cardiovascular surgery. However, GA treatment causes tissue calcification. No rapid anticalcification method has been established for use during surgery. Here, we aimed to establish a rapid anticalcification method using ethanol, as has already been demonstrated for bioprosthetic valves.

**Methods:**

Thoracic aorta tissues were first fixed with GA for 3 min and then treated with ethanol for 0 (group 2), 10 (group 3), 20 (group 4), and 30 (group 5) min; untreated tissues (group 1) served as the control. The treated tissues were subdermally implanted into 3-week-old male Wistar rats and kept in place for 28 days. The calcification in each explant was semiquantitatively evaluated by annotating and measuring the area using virtual slides, and the data obtained were statistically analyzed.

**Results:**

Semiquantitative analysis revealed that calcification of the implants from the untreated group (group 1; *P* = 0.0014) and groups 4 (*P* = 0.0014) and 5 (*P* = 0.0031) was significantly lower than that of implants from group 2. Moreover, implants from group 3 showed a tendency toward decreased calcification, although it was not significant (*P* = 0.0503).

**Conclusions:**

A rapid ethanol treatment prevents calcification of GA-fixed tissues in a rat model of subdermal implantation. This method may facilitate effective and rapid anticalcification of autologous tissues for use during cardiovascular surgery.

**Supplementary Information:**

The online version contains supplementary material available at 10.1186/s13019-022-01895-7.

## Background

The prevalence of cardiovascular disease, a leading cause of morbidity and mortality, has been increasing worldwide. Autologous tissues, including the pericardium, are widely used as patches and valve substitutes in cardiovascular surgery [1]. In addition to fresh tissue, glutaraldehyde (GA)-fixed tissues are used owing to their tensile strength and amenability to better handling. The use of an autologous pericardium for aortic valve plasty was first reported by Durán et al. in 1988 [2]. A decade later, aortic valve neocuspidization (the Ozaki procedure), a novel surgical procedure to replace aortic valve cusps by a GA-treated autologous pericardium, was developed [3]. However, GA-fixed autologous tissues are prone to calcification after long-term implantation, which limits their durability [4]. Calcification is induced by several factors, including tissue phospholipids and free aldehyde groups of GA [5]. Various anticalcification methods have been proposed for bioprosthetic valves [6], but in most of the methods, the treated tissue can only be used after several days. For instance, Vyavahare et al. [7, 8] employed a 24 h ethanol treatment to reduce phospholipid content. However, no rapid anticalcification method that can be used in the operating room within a limited time during a surgical procedure has been reported.

We hypothesized that ethanol may be applied for rapid anticalcification, as has already been demonstrated for bioprosthetic valves. The objective of this study was to establish a rapid anticalcification method using ethanol.

## Methods

### Tissue preparation

The donor rats were euthanized via CO_2_ inhalation. The thoracic aorta was excised from the donor rats and divided into five groups according to the method of tissue preparation. For decalcification, 0.625% GA in phosphate-buffered saline (PBS; 2% glutaraldehyde fixative [Muto Pure Chemicals Co., Ltd, Tokyo, Japan] in 100 mL of PBS [pH 7.4; Gibco, NY, USA]) and 80% ethanol (400 mL of 99.5% ethanol [FUJIFILM Wako Pure Chemical Corporation, Osaka, Japan] in 100 mL of distilled water) were used [7, 9]. Group 1 contained untreated tissues as the control; group 2 contained tissues fixed with GA for 3 min; group 3 contained tissues fixed with GA for 3 min and post-treated with ethanol for 10 min; group 4 contained tissues fixed with GA for 3 min and post-treated with ethanol for 20 min; and group 5 contained tissues fixed with GA for 3 min and post-treated with ethanol for 30 min. The samples were fixed at room temperature and rinsed in normal saline to remove residual GA before implantation.

### Rat model of subdermal embedding

Eleven 3-week-old male Wistar rats (Oriental Yeast Co., Ltd.) were used as recipients. The rats were anesthetized via the inhalation of isoflurane maintained at 2–3%, after which the hair on each rat was shaved, and dorsal subdermal pouches were created. The treated tissue samples were implanted, and the wounds were closed with 5-0 polypropylene suture (Ethicon Inc., Somerville, NJ, USA). This subdermal implantation model is a rapid and reliable tool for evaluating the effects of anticalcification on tissues [10]. Each rat was maintained and observed in its own cage after surgery. The rats were provided dry food and water ad libitum through an automatic watering system. The housing rooms of the rats were maintained at 24 °C ± 1 °C and relative humidity between 50 and 60% under a 12:12 h photoperiod. Ceftriaxone sodium hydrate (0.3 g) was subcutaneously injected into the rats post-operation. After 28 d, the rats were euthanized via CO_2_ inhalation, and the implanted tissues were harvested and rinsed with normal saline. All tissue samples were immediately stored in 10% buffered formalin. All rats survived the implantation and experimental period.

### Histological processing of the explants

Formalin-fixed explants were embedded in paraffin, cut into 5 µm-thick sections, and stained with hematoxylin and eosin (H&E) and Victoria blue (VB) for histological analysis. The slides were examined by light microscopy and evaluated by pathologists, blinded to the grouping, for calcification, granulation, and host inflammatory response.

### Tissue calcification and granulation analysis

**Fig. 1:**
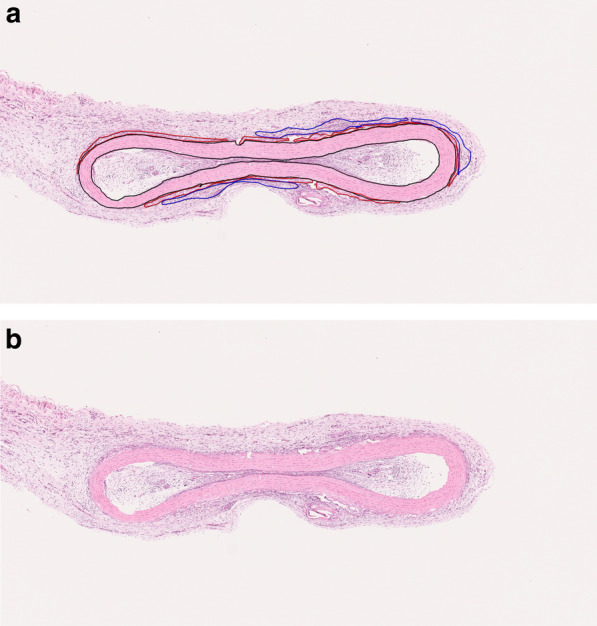
Annotation and representative histological features of the thoracic aorta. **a** Entire tissue area (black line), granulated tissue (blue line), and extent of calcification (red line) were annotated for each sample of thoracic aorta; area was measured in mm.^2^ and evaluated semiquantitatively. **b** Representative histological features of the explanted thoracic aorta. Granulation and calcification covered the tissue surface (stain, H&E; magnification, × 5)

The stained slides were scanned and digitally converted into virtual slides using a Hamamatsu NDP slide scanner (Hamamatsu NanoZoomer 2.0-HT; Hamamatsu Photonics, Hamamatsu, Japan). The virtual slides were viewed and analyzed using NDP.View2 (Hamamatsu Photonics). The calcification and granulation levels in each sample were semiquantitatively evaluated by annotating and measuring the visual area (Fig. 1a).

### Statistical analysis

Data were analyzed using EZR for Windows (Saitama Medical Center, Jichi Medical University, Saitama, Japan). Analysis of variance was used for multiple comparisons among different time points. For paired comparisons, the *t*-test and Kruskal–Wallis test were used to detect differences between the experimental groups. Statistical significance was set at *P* < 0.05. All data are expressed as mean ± standard deviation.

## Results

### Microscopic examination (pre-implantation)

Histologically, all pre-implantation tissues showed optimal preservation of the elastic arterial media without any inflammatory changes.

### Microscopic examination (post-implantation)

**Fig. 2:**
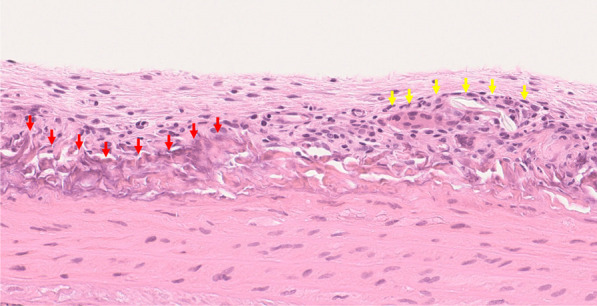
Tissue calcification and granulation. Explanted thoracic aorta was covered with granulated tissue composed of fibroblasts, capillary vessels, and inflammatory cells, such as lymphocytes and macrophages (yellow arrows). Collagen fibers of the adventitia were degenerated and considered calcified (red arrows) (stain, H&E; magnification, × 30)

**Fig. 3:**
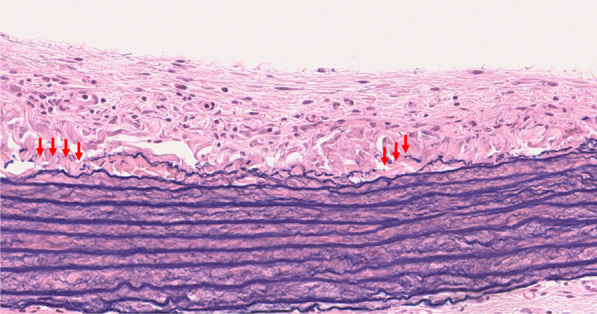
Changes in the tunica media. Microscopy findings showing almost preserved elastic arterial media. In areas with noticeable calcification, the outermost elastic fibers of the tunica media have disappeared (red arrows) (stain, VB; magnification, × 30)

Histologically, the surface of each explant was covered with granulated tissue and was calcified to different degrees (Fig. 1b). At the periphery of the arterial wall, calcification, concomitant with collagen fiber degeneration, was observed (Fig. 2). Elastic fibers in the tunica media were almost preserved; however, those in the outermost layer of tunica media had slightly disappeared (Fig. 3).

### Analysis of calcification

**Fig. 4:**
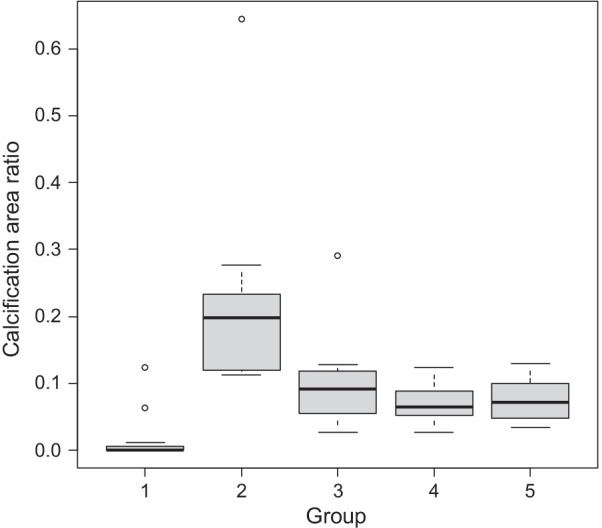
Semiquantitative analysis of calcification. Group 1 (control): untreated tissues; group 2: tissues fixed with glutaraldehyde (GA) for 3 min; group 3: tissues fixed with GA for 3 min and post-treated with ethanol for 10 min; group 4: tissues fixed with GA for 3 min and post-treated with ethanol for 20 min; group 5: tissues fixed with GA for 3 min and post-treated with ethanol for 30 min. Semiquantitative analysis of calcification revealed the calcified areas in the explants of groups 1, 2, 3, 4, and 5 to be 0.02 ± 0.04, 0.22 ± 0.15, 0.10 ± 0.07, 0.07 ± 0.03, and 0.08 ± 0.03 mm^2^, respectively. Calcification of explants from groups 1 (*P* = 0.0014), 4 (*P* = 0.0014), and 5 (*P* = 0.0031) was significantly lower than that of explants from group 2. Group 3 (*P* = 0.0503) showed slightly decreased calcification although the decrease was not statistically significant. Calcified areas are displayed as a bow-and-whiskers plot with median (thick line), 25th to 75th percentiles (box), 10th to 90th percentiles (whiskers), and outliers (circles). *P*-values were calculated using the Kruskal–Wallis test

Semiquantitative analysis of calcification revealed that the calcified areas in the explants of groups 1, 2, 3, 4, and 5 were 0.02 ± 0.04, 0.22 ± 0.15, 0.10 ± 0.07, 0.07 ± 0.03, and 0.08 ± 0.03 mm^2^, respectively (Fig. 4). The calcified areas in the explants of groups 1 (*P* = 0.0014), 3 (*P* = 0.0503), 4 (*P* = 0.0014), and 5 (*P* = 0.0031) were significantly lower than those in group 2.

### Analysis of granulated tissues

A few granulated tissues were observed in group 1 (0.20 ± 0.16 mm^2^). More granulated tissues were observed in the other groups than in group 1, and the extent of granulation was comparable among groups 2 (0.33 ± 0.16 mm^2^), 3 (0.28 ± 0.13 mm^2^), 4 (0.33 ± 0.16 mm^2^), and 5 (0.26 ± 0.08 mm^2^), as revealed in the semiquantitative analysis. The granulation areas in the explants were not significantly different from those in the explants of group 2 (Additional file 1: Fig. S1).

## Discussion

The present study was aimed at evaluating a rapid anticalcification method for GA-fixed autologous tissues. The major finding was that calcification was significantly lower in autologous tissues fixed with GA for 3 min and post-treated with ethanol for 20–30 min than that in autologous tissues without ethanol post-treatment. These results suggest that rapid ethanol treatment exerts anticalcification effects similar to long-time treatment, which has already been demonstrated for bioprosthetic valves.

To the best of our knowledge, this is the first report on the anticalcification efficacy of rapid ethanol treatment in a rat subdermal implantation model. Vyavahare et al. [7, 8] reported that preincubation of GA-fixed porcine aortic valve bioprostheses in ethanol prevented cuspal calcification in vivo. They showed that the effects of extracting phospholipids and cholesterol and alteration in collagen conformation are parts of the mechanism for preventing calcification via ethanol pretreatment. Although the mechanism of calcification was not elucidated in the present study and the processing time was only shortened, we believe that the mechanism might not be much different. In the absence of literature on the processing time for rapid ethanol pretreatment, we explored different durations (1, 3, 5, 10, 20, and 30 min). However, because we obtained meaningful results for time points beyond 10 min, we have not presented the results obtained for 1, 3, and 5 min treatments. Although the optimal processing time is unclear, we show that 20–30 min of ethanol treatment reduces the calcification level of GA-fixed tissues. Although statistically significant results were not obtained for the 10 min ethanol treatment, a tendency toward decreased calcification was observed. Notably, the expected trend of decreasing calcification with increasing time of ethanol treatment was not significant. This may be due to various reasons. First, the sample size was too small for analysis. Second, the difference might not have been evident for 10 min intervals because in the long-term ethanol treatment used in previous studies, 1 day intervals were employed. As mentioned previously, Vyavahare et al. [7] showed the effect of ethanol pretreatment for 1–3 d. Similarly, Lee et al. [11] reported the effect of treatment with glycine, another anticalcification agent, for 1–3 d. Lastly, although calcification was reduced to some extent by a 10 min ethanol treatment, most changes might have occurred in the first dozen minutes; that is, considerable phospholipids may have been removed during this time. Further studies are required to examine the relationship between ethanol treatment time and the calcification and phospholipid levels.

Inflammation and immune responses play important roles in calcification [12, 13]. As autologous pericardium elicits lower inflammation and immune response than heterologous pericardium, it is considered a better material for patches and valve substitutes in cardiovascular surgery [14]. In this study, although we show that rapid ethanol treatment exerts anticalcification effects, the level of granulation did not reach statistical significance with ethanol treatment, suggesting that rapid ethanol treatment for anticalcification did not promote further inflammatory reaction. Our findings are consistent with those of a previous study [7]. Ethanol treatment only inhibited calcification and prevented the promotion of the inflammatory reaction. These findings indicate that ethanol treatment may be an effective and safe method for anticalcification.

Although the results of the present study are promising, there are some limitations to the study. First, the changes observed in the rat model may differ from those in the blood contact model of circulatory conditions but not subdermal conditions. In addition, the treatment was only for 28 days, and we did not use pericardium because it was too thin to examine. Second, calcification was evaluated semiquantitatively, and not quantitatively. Third, the sample size was too small for analysis, and therefore, this study could only be considered a pilot study for examining the efficacy of anticalcification treatment. Finally, although the efficacy of anticalcification effects of brief ethanol treatment is suggested, the function and durability of the tissue were not evaluated.

Future studies should focus on identifying optimal processing times for GA and ethanol from the perspective of calcification and material characteristics, such as durability using a long-term circulatory model, with a large sample size.

## Conclusion

We provide evidence for the anticalcification effects of brief ethanol treatment similar to those of long-time treatment. Further studies are required to examine the anticalcification effects of pericardium in a large animal blood contact model and the effect of brief ethanol treatment on explant characteristics.

## Supplementary Information

**Additional file 1**. Semiquantitative analysis of granulation.

## Data Availability

All data generated or analyzed during this study are included in this published article and its supplementary information files.

## Abbreviations

GA: Glutaraldehyde
PBS: Phosphate-buffered saline
H&E: Hematoxylin and eosin
VB: Victoria blue
