# Supplementary figures and images for: Rapid anticalcification treatment for glutaraldehyde-fixed autologous tissue in cardiovascular surgery
Source: J Cardiothorac Surg. 2022 May 31;17:138. doi: 10.1186/s13019-022-01895-7 (PMC9158145; doi:10.1186/s13019-022-01895-7)

## Slide 1
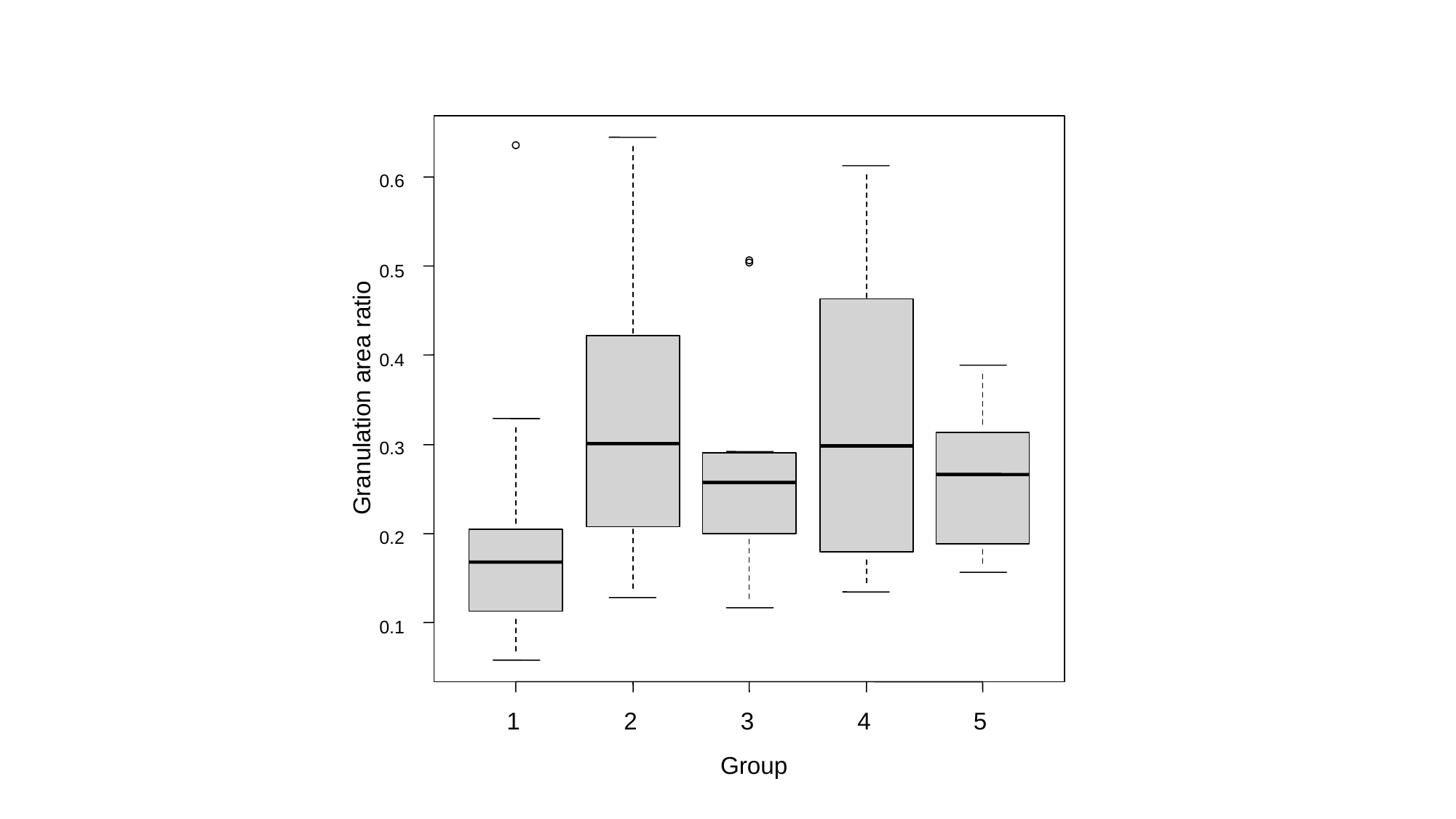

0.6
0.5
0.4
Granulation area ratio
0.3
0.2
0.1
1
2
3
4
5
Group

Supplement: Supplementary file 1 — Additional file 1. Semiquantitative analysis of granulation. [file 13019_2022_1895_MOESM1_ESM.pptx]
